# *“What you don’t know won’t harm you”:* An interpretative phenomenological analysis of barriers and facilitators of poor early breast cancer screening uptake among black women in England

**DOI:** 10.3389/fonc.2026.1875824

**Published:** 2026-07-01

**Authors:** Tarela Juliet Ike, Dung Ezekiel Jidong, Mieyebi Lawrence Ike, Evangelyn Ebi Ayobi, Peremi Richmond Ike, Rukevwe Francis Doghor, Oluwakorede Florence Adebowale, Gabriella Rooks, Nusrat Husain

**Affiliations:** 1 Division of Psychology and Mental Health, University of Manchester, Manchester, United Kingdom; 2 School of Social Sciences, Humanities and Law, Teesside University, Middlesbrough, United Kingdom; 3 Western Governors University, Millcreek, UT, United States; 4 Manchester Global Foundation, London, United Kingdom; 5 Tare Wyd Legal Chambers, Warri, Delta, Nigeria; 6 Beryl Foundation, London, United Kingdom; 7 Moonrise 24 Hrs Recruitment, Stockton-on-Tees, United Kingdom; 8 Mersey Care National Health Service (NHS) Foundation Trust, Prescot, United Kingdom

**Keywords:** black women, breast cancer, early screening uptake, England, United Kingdom

## Abstract

**Background:**

Breast cancer constitutes a global public health concern. However, Black women in the United Kingdom (UK) experience disproportionately low breast cancer screening uptake despite higher risks of late−stage diagnosis and poorer outcomes. This highlights a significant gap, and the study addresses this gap by making an original contribution using interpretative phenomenological analysis (IPA) to explore the lived experiences of Black women to understand the barriers and facilitators of poor early breast cancer screening uptake in the United Kingdom.

**Methods:**

One focus group comprising 10 participants and 5 in−depth semi−structured interviews were conducted with Black women living in England, and the data were analysed using IPA.

**Findings:**

The study found that cultural modesty, stigma, fear, and fatalistic beliefs intersect to shape vulnerability and perceptions of screening. It also found that trauma, mistrust, structural disadvantages such as unequal access to healthcare and past negative healthcare experiences further serve to negotiate and sustain beliefs around screening.

**Conclusion:**

This study provides a nuanced understanding with significant policy relevance by demonstrating and recommending the need for tailored messages, trauma−informed, and psychosocially supportive screening pathways as s essential for reducing inequalities and ensuring that screening services are accessible, respectful, and emotionally safe for Black women.

## Introduction

Breast cancer constitutes a global disease burden that affects an estimated 2.3 million women diagnosed in 2022 alone (1). It also amounts to 670,000 deaths, thus representing a leading cause of mortality among women globally (1). In the United Kingdom (UK), breast cancer is one of the most common cancers, with data from the National Institute for Health and Care Excellence (2) suggesting that an estimated 1 in 7 women are diagnosed during their lifetime. In addition, in the UK, over 56, 000 women are diagnosed annually, with 80% of cases occurring in women above the age of 50 (3). Of central concern are Black women who experience disproportionate effects of breast cancer in the UK. Data from Breast Cancer Now (4) suggest that Black African women in the UK are less likely to go to their breast screening appointment, with only an estimated 49% of Black African women who are invited to breast screening take up the screening appointment, compared to 67% of white women (4).

Yet persistent inequalities in screening uptake continue to place Black women at heightened risk of late−stage diagnosis and poorer outcomes. In the UK, Black women, comprising Black African and Caribbean women, are twice as likely to be diagnosed with high-grade, late-stage breast cancer compared to white women (5). Black women in the UK are also more likely to present with aggressive tumour subtypes and at more advanced stages, yet they consistently demonstrate lower participation in routine breast cancer screening programmes (6). Existing literature attributes these disparities to a complex interplay of misconceptions and poor understanding of the causes of breast cancer (7, 8) cultural beliefs (9), emotional responses, structural inequities such as racism, and historical mistrust in the GP and health care settings (9), underscoring the need for research that centres Black women’s lived experiences to understand the barriers to early breast cancer screening uptake.

A substantial body of literature highlights the role of cultural beliefs and norms in shaping screening behaviour (6, 9). Studies have shown that cultural modesty, stigma surrounding breast exposure, and fatalistic beliefs about cancer are particularly salient among African and African diaspora women (10, 11). These beliefs often frame cancer as an inevitable or spiritually determined outcome, reducing perceived benefits of early detection. However, much of this literature treats culture as a static barrier rather than a dynamic meaning−making system. In addition, there remains limited qualitative work exploring how Black women interpret these cultural norms in their everyday lives.

Communication and health messaging also play a critical role in shaping screening engagement. Evidence from countries including the United States (12), Canada (13), and Chinese women in America (14) suggests that tailored interventions that reflect community values, use relatable narratives, and are delivered through trusted channels significantly improve screening uptake among minority groups. Narrative−based communication has been shown to reduce fear, enhance emotional resonance, and counteract fatalistic beliefs (15). Yet mainstream UK screening campaigns often adopt an approach that fails to acknowledge cultural diversity or address the specific concerns of Black women. This gap highlights the need for research that examines how Black women face the barriers to early breast cancer screening and what forms of messaging they find meaningful and trustworthy.

Another critical dimension in the literature concerns mistrust and trauma. Research consistently documents that Black women in the UK experience poorer communication, reduced continuity of care, and higher rates of negative encounters within the NHS, particularly in maternity and reproductive health services (16). These experiences appear to contribute to a broader pattern of medical mistrust that extends into cancer screening contexts. Trauma-informed care has been proposed as a framework for addressing these issues, emphasising emotional safety, transparency, and sensitivity to past harm (17). However, there is limited empirical work exploring how trauma and mistrust specifically shape Black women’s decisions about breast cancer screening, and even less that examines how these experiences intersect with cultural identity and systemic disadvantage.

Structural inequities further compound these challenges, and this include socioeconomic disadvantage, limited access to culturally competent care, and systemic racism within healthcare institutions that have all been identified as barriers to screening (18). There is a growing recognition that understanding screening disparities requires moving beyond surface-level explanations to explore how Black women make sense of their experiences within broader social and historical contexts.

Despite these important contributions, significant gaps remain, especially as it relates to employing methodologies such as Interpretative Phenomenological Analytical (IPA) lens capable of capturing the complexity, nuance, and emotional depth of Black women’s experiences. IPA offers a valuable approach for addressing this gap by foregrounding lived experience, exploring meaning-making processes, and attending to the interplay between personal, cultural, and structural factors such as racism, poor access to health care and socioeconomic barriers.

This study builds on and extends the existing literature by using IPA to explore how Black women in the UK understand and navigate the barriers to breast cancer screening in England. By centring their voices, the study provides a richer, more nuanced understanding of the factors shaping screening behaviour and highlights the need for culturally responsive, trauma-informed, and psychosocially supportive screening pathways. The use of Interpretative Phenomenological Analysis provides methodological rigour in that it offers a uniquely powerful and original lens for exploring Black women’s experiences of poor breast cancer screening uptake in England because it prioritises the depth, nuance, and emotional complexity that quantitative studies often overlook. IPA’s commitment to understanding how individuals make sense of their experiences (8) allows for a rich examination of how cultural beliefs, trauma, mistrust, and structural inequities such as unequal access to care shape screening behaviour in ways that cannot be captured through surface-level measures. Its idiographic focus is particularly significant in this context, as Black women’s perspectives have historically been marginalised within UK health research, resulting in a limited understanding of how screening avoidance is lived and felt. By engaging closely with participants’ meaning-making processes, IPA enables the illumination of the interplay between personal history, cultural identity, and systemic disadvantage, offering insights that challenge dominant narratives and reveal the psychological realities behind persistent screening inequalities.

Against the preceding backdrop the study addressed the following research question below:

- What are Black women who are partially or not up to date with breast cancer screening experiences of the barriers to early screening uptake in the UK?

For this study, partially refers to having had one of the screenings done but not completed full screening. To address the research question, the next section details the methodology adopted. This is followed by the findings and a discussion linked to the literature.

## Methodology

The present study is informed by a case study design, underpinned by an interpretative epistemology and a constructionist ontology (19), which offers a significant framework for exploring Black women’s lived experiences of poor breast cancer screening uptake in England. The fusion of interpretative epistemology (19) and constructionist ontology (20) approach recognises that reality is not fixed but subjective, constructed and interpreted through social, cultural, and historical contexts, making it well−suited to examining how Black women interpret and give meaning to screening within their everyday lives. By treating Black women’s experiences as situated, context−dependent cases rather than generalisable data points, the study is able to capture the complexity of cultural norms, trauma, mistrust, and inequities that shape screening behaviour. The interpretative stance allows engagement with participants’ in-depth meaning-making processes, while the constructionist ontology acknowledges that these meanings are shaped through interactions with healthcare systems, communities, and wider societal narratives. Together, this methodological alignment provides a rigorous and original lens for understanding the nuanced barriers that contribute to screening disparities. The methodological alignment, coupled with the use of Eatough and Smith’s (8) Interpretative Phenomenological approach, also provides robust analytical insight into the participants’ lived experiences of the barriers to poor early breast cancer screening uptake.

### Ethics

Prior to data collection, ethical approval was sought from and granted by the Teesside University Research Ethics sub-Committee. This study was also conducted in strict accordance with ethical principles outlined in the Declaration of Helsinki, ensuring that participants’ dignity, rights, and welfare were prioritised throughout the research process. Informed consent was obtained from all participants, who were fully briefed on the study aims, procedures, and their right to withdraw without consequence. Given the sensitivity of discussing trauma, mistrust, and healthcare experiences, particular care was taken to ensure confidentiality, emotional safety, and access to appropriate support if needed. Prior to participation, all participants were briefed on the study’s purposes and provided with the participant information sheet. The participant was also briefed on how their study data will be used, including for publication, and on their right to withdraw without being obliged to give reasons, including the grounds for disclosure. All participants duly consented by completing the electronic consent form prior to taking part in the study.

### Participant demographics and sampling

A purposive sampling strategy was used to recruit participants whose experiences were directly relevant to the study aims. Recruitment was facilitated through established relationships with third−sector organisations in person and via telephone, including non−governmental and faith−based groups such as churches and mosques, which provided access to women embedded within the communities of interest. To broaden reach and ensure inclusion of individuals with comparable lived experiences, participants also recommended others who might be willing to take part. This approach enabled the development of a focused yet diverse sample while preserving the intentional and theoretically driven nature of purposive sampling. A total of 21 prospective participants were approached for the study and six declined to participate citing personal reasons they do not wish to disclose as the rationale.

In total, 15 females with lived experiences of non or partial breast cancer screening were recruited for the study. Of the 15, n=3 had attained a secondary level of education, while the other 12 indicated a tertiary level of education. Regarding employment status, 11 were employed, while the other 4 indicated being unemployed. The participants were recruited from England and comprised regions such as Middlesbrough, Redcar and Cleveland, Manchester, Hartlepool, and the Tees Valley area. Of the 15 participants, 12 identified as being from African countries, including Nigeria (n=5), Ghana (n=2), South Africa (n=3), Kenya (n=1), and Somalia (n=1), while the other 3 identified as being from Caribbean countries, including Jamaica, Trinidad and Tobago, and Barbados. All participants were first-generation migrants. The participants were between 18 and 65 years old.

### Data collection

A total of 15 participants were recruited for the study. One focus group comprising 10 participants and 5 individual interviews were conducted. Using both focus groups and individual interviews provides a complementary approach that aligns with IPA’s commitment to capturing rich, detailed accounts of lived experience. Focus groups enable shared cultural meanings and collective narratives to surface—particularly valuable when exploring community-embedded issues such as screening stigma, mistrust and issues around cancer-related topics (21, 22)—while individual interviews allow for deeper exploration of personal, sensitive, or trauma-related experiences that participants may not disclose in a group setting (23). Combining these methods strengthens the interpretative depth of the analysis and supports a more nuanced understanding of the sociocultural and emotional barriers shaping Black women’s engagement with breast cancer screening (24). The data was collected virtually via Microsoft Teams and recorded. The focus group lasted approximately 90 minutes, while the individual interviews lasted, on average, 1 hour. The interviews and focus group were conducted by a Black woman with lived experience who had a PhD-level qualification. Data saturation was attained at the fifth interview, at which no new themes emerged; similar findings from the focus group were echoed in the individual interviews.

### Data analysis

The recorded individual interviews and focus group discussions were transcribed verbatim into a Word document and uploaded into NVivo version 15 for data analysis. The data were analysed using the systematic, iterative stages of Interpretative Phenomenological Analysis (8), beginning with repeated, immersive readings of each transcript to develop a close familiarity with the participant’s account. Initial exploratory notes were made on descriptive content, linguistic features, and conceptual reflections, which then informed the development of emergent themes grounded in the participant’s meaning−making. These themes were subsequently clustered into higher−order experiential patterns, while maintaining a strong commitment to ideography by analysing each case in depth before moving to the next. Only after each transcript had been individually interpreted were patterns of convergence and divergence examined across cases, allowing for a nuanced, layered understanding of the shared and unique elements of Black women’s experiences of breast cancer screening. IPA as an analytical lens have also been used in other sensitive topic areas, including victims affected by trauma (25), terrorism (26) and mental health (27).

To ensure analytic rigour and enhance intercoder reliability, two members of the research team with lived experience of breast cancer screening, trained in conducting interpretative phenomenological analysis, independently reviewed a subset of transcripts and the corresponding emergent themes. This was further reviewed by two senior team members with doctoral-level qualifications (e.g. PhD) to ensure consistency. Differences in interpretation were discussed collaboratively, not to force consensus, but to deepen reflexive engagement and strengthen the credibility of the analytic process in line with IPA’s interpretative stance. Regular reflexive meetings were held to examine assumptions, challenge potential biases, and ensure that interpretations remained grounded in participants’ lived experiences rather than the research team’s preconceptions. This combination of independent review, reflexive dialogue, and transparent decision−making supported a robust and trustworthy analytic process while honouring the interpretative depth central to IPA. Below is an example of a table summarising themes and representative quotes (**Table 1**).

**Table 1:** Sample summary themes and representative quotes.

Transcripts	Emergent themes	Clustered superordinate themes
“For me, I would say religious barrier because, all right, we just hope in God because he is the healer [ … ]. So, we believe anything, we take it up to God. And also, social barrier in the sense that, okay, if there’s no symptoms, I just feel it’s not necessary, right, to go for the screening. All right, so if there’s no, visible symptoms in a way. So that has, which is not, it shouldn’t be an excuse, but because of lack of awareness of what we should do in advance, so it has limits or is one of the barriers that limits breast cancer screening.” (Ashantae – Focus Group). “I don’t even want to hear about it. So just pretend as if it’s not there and you are safe. And also, the spiritual aspects [ … ] As long as they are a child of God, when you pray, everything will be fine and it can be cured.” (Kwashi – Focus Group)	- Reliance on religious beliefs - Symptom_driven decisions - visible signs as triggers	Faith-driven reassurance and chosen unawareness
“Media campaign intervention is something that I will feel comfortable to encourage me to engage in early breast cancer screening uptake. The campaign intervention can target radio or social media platforms but is encouraged to reflect the cultural values of us as Africans, including our concerns and fears and the procedures to go for breast cancer screening to encourage our uptake” (Tanashae – Individual Interview) “The media is a formidable force that people listen to, and if it is used effectively by capturing, for example, my culture and religious beliefs as a Black person and woman, I think it can definitely sway me to go for breast cancer screening.” (Felina – Focus Group)	- Media - Culturally appropriate content - Influence on screening uptake	Tailored communication as a catalyst for screening uptake

### Findings

Based on analysis drawing on an interpretative phenomenological analytical lens the following main superordinate themes emerged including (i) Faith-driven reassurance and chosen unawareness, (ii) Stigma, anxiety and adverse social visibility as barriers to screening, (iii) Cultural modesty, sensitivity and fatalistic beliefs as barriers to screening, (iv) Tailored communication as a catalyst for screening uptake and (v) Trauma, mistrust, and call for psychosocially safe screening pathways.

### Faith-driven reassurance and chosen unawareness

A dominant narrative expressed in the participants’ lived experiences was how individuals appear to use both deliberate avoidance of illness−related information and strong spiritual beliefs as psychological shields against the fear of breast cancer. By embracing the idea that the unknown cannot cause harm and by relying on faith for protection and healing, participants create a sense of emotional safety that reduces the perceived need for screening. This protective view appears to offer comfort but ultimately limits engagement with preventive health behaviours. Talking about this, one participant said:

For me, I would say religious barrier because, all right, we just hope in God because he is the healer [ … ]. So, we believe anything, we take it up to God. And also, social barrier in the sense that, okay, if there’s no symptoms, I just feel it’s not necessary, right, to go for the screening. All right, so if there’s no, visible symptoms in a way. So that has, which is not, it shouldn’t be an excuse, but because of lack of awareness of what we should do in advance, so it has limits or is one of the barriers that limits breast cancer screening. (Ashante – Focus Group).

The participant’s account reveals a complex interplay between faith, symptom-based health beliefs, and limited awareness, all of which shape her engagement with breast cancer screening. Her reliance on God as *“the healer”* positions spiritual trust as a primary coping mechanism, offering reassurance while simultaneously reducing the perceived need for medical intervention. In making sense of the participant’s lived experience, this view appeared reinforced by a belief that illness is only meaningful when physically visible, leading her to view screening as unnecessary *“if there’s no symptoms,”* which reflects a reactive rather than preventive understanding of health. Yet, the participant also demonstrates emerging self-awareness, acknowledging that this reasoning “shouldn’t be an excuse” and recognising that a *“lack of awareness”* contributes to screening avoidance. This tension suggests that participants are to be negotiating between deeply rooted cultural and religious frameworks and growing knowledge about preventive health, highlighting an internal struggle between comfort in familiar beliefs and the demands of proactive medical practice. Another participant also notes:

There is a general belief that what you don’t know won’t harm you. So, to be on the safer side, most people tend to avoid the topic completely. They don’t even want to hear about it. So just pretend as if it’s not there and you are safe. And also, the spiritual aspects, people, including myself believe in, okay, there’s nothing like that. As long as they are a child of God, when you pray, everything will be fine, and it can be cured. (Kwashi – Focus Group)

The participant’s account reflects a deeply rooted cultural belief that avoiding knowledge about illness offers psychological protection, encapsulated in the idea that *“what you don’t know won’t harm you.”* This belief appears to function as a coping strategy, allowing the participant to distance herself from the fear and uncertainty associated with breast cancer by *“pretending as if it’s not there.”* Alongside this avoidance, the participant highlights a powerful spiritual framework in which faith provides a sense of immunity, suggesting that being *“a child of God”* and praying ensures safety and healing. This spiritual confidence appears to offer emotional reassurance but simultaneously diminishes the perceived relevance of medical information or screening. The narrative reveals an interplay between fear−driven avoidance and faith−based optimism, both of which shape how the participant and their community make sense of health risks. Ultimately, the account illustrates how cultural narratives and spiritual beliefs converge to create a protective yet limiting view that reduces engagement with preventive health behaviours, such as early screening.

### Stigma, anxiety and adverse social visibility as barriers to screening

Navigating the decision to engage in early breast cancer screening uptake can be challenging, especially following perceived societal reaction in the process. A notable pattern that resonates across most participants’ lived experiences was how the fear of being judged, labelled, or socially avoided becomes a significant psychological barrier to breast cancer screening. Participants describe living with the anticipation of stigma—imagining others treating them differently—which transforms screening from a private health decision into a public risk to identity. Within close-knit social environments where information spreads quickly, the possibility of being marked as someone associated with illness generates anxiety, leading individuals to avoid screening to protect their social standing, sense of normality, and belonging. Commenting on the issue, one participant said:

So, for me, I would talk about the social barrier that has to do with stigma. You know, when you live in a close cycle where information spreads really fast and when people get to, oh, this is what is actually wrong with this person, people tend to avoid such person. (Chisom – Focus group)

As the preceding extract shows, the participant’s account reveals a deeply personal experience of stigma as a powerful social barrier, shaped by the dynamics of living in a *“closed cycle”* where information circulates rapidly and judgment is swift. Phenomenologically, the participant describes the embodied anxiety of being scrutinised and potentially avoided, highlighting how the fear of social exclusion becomes intertwined with the experience of illness itself. Through a hermeneutic lens, the participant’s narrative reflects an interpretative struggle by making sense of stigma not merely as external behaviour but as a threat to identity and belonging, interpreting avoidance by others as a form of moral or social contamination. In making sense of the participant’s lived experience, she therefore appears to situate this meaning within the community context, emphasising how tightly knit social environments intensify the consequences of disclosure. The participant account illustrates how the anticipation of being labelled or ostracised shapes health−related decisions, revealing stigma as not just a social reaction but a lived, internalised barrier that profoundly influences how individuals navigate vulnerability and engagement in early breast cancer screening uptake. On a slightly similar note, another participant also said that:

The fear too is a big issue and stigma of how you will be treated in the society. I don’t want people to be looking at me weird that oh see that lady she went to screen for breast cancer and then they just start treating you somehow and avoiding you (Tanashae – Individual Interview)

The participant’s account conveys a deeply felt fear of social judgment, describing stigma as an ever−present threat that shapes how they navigate the possibility of breast cancer screening. Phenomenologically, the participant articulates the emotional weight of imagining others *“looking at me weird”* or treating her *“somehow,”* revealing how the anticipation of being viewed differently becomes as distressing as the illness itself. Through a hermeneutic lens, her narrative reflects an interpretative struggle in which screening is not simply a medical act but a symbolic marker that others may read as evidence of illness, weakness, or contamination, prompting avoidance and social distancing. In essence, the participant situates this meaning within her own social world, one in which community reactions carry significant personal consequences and in which being labelled can alter everyday interactions. The participant account illustrates how the fear of altered social identity—of becoming *“that lady”* who went for screening—creates a powerful psychological barrier, showing that stigma is not merely external judgement but a lived, internalised experience that shapes health behaviour long before any diagnosis occurs.

### Cultural modesty, sensitivity and fatalistic beliefs as barriers to screening

This theme captures how deeply rooted cultural norms around bodily privacy, modesty, and the fear of exposing intimate parts of the body create significant emotional barriers to breast cancer screening. Bodily privacy was construed from the perspective of cultural norms of perceived decency. Participants describe how beliefs from African and specifically, Nigerian cultural contexts—such as the taboo of revealing the breast and the perception of cancer as a “death sentence”—shape their understanding of screening as frightening, shame-laden, and potentially harmful. Even after migrating to the UK, these cultural scripts persist, generating fear, avoidance, and a sense that screening threatens both emotional well-being and cultural identity. Commenting on this, one participant said:

Culturally, I think it’s not something that is discussed openly. In some Black communities, like topics like breast cancer can feel private or even uncomfortable to really talk about, especially with strangers or with male doctors. So, I think that culturally, that can really stop women from going for screening, including myself. (Layefa – Individual interview)

The participant’s account highlights how cultural norms around privacy and discomfort profoundly shape the lived experience of breast cancer screening, particularly within Black communities where such topics *“are not discussed openly.”* Phenomenologically, the participant describes a sense of unease and vulnerability that arises when intimate health concerns intersect with social expectations, especially when disclosure involves “*strangers or male doctors,”* whose presence intensifies feelings of exposure. Through a hermeneutic lens, the participant interprets this cultural silence as both a protective mechanism and a barrier, revealing how deeply embedded norms around modesty, privacy, and propriety influence her understanding of what is acceptable to share. As such, the participant’s narrative appears rooted in cultural context, showing how these collective values shape her personal hesitation and contribute to a broader pattern of avoidance within the extant community. Ultimately, the account illustrates how cultural discomfort and gendered dynamics create a significant emotional and social landscape that discourages screening, because the act itself appears to feel culturally transgressive and personally exposing. Another participant also said:

The breast being a very private part of the body, in terms of culture, just like from Africa, like specifically from Nigeria, we have like cultural beliefs in terms of like, not exposing your breast, even to the person that’s going to even examine, you know, that’s going to examine the patient, you know. So that’s here. And again, also the word cancer, everybody believes that once anyone has cancer or once anyone has been diagnosed of cancer means like a death sentence, which is very, very scary to everyone to even want to participate in the screening [ … ]. Because if they are aware that they have been diagnosed of cancer, they could be depressed, which could cause like an early death for them. but even the diagnosis killed the person. So really, really like a concern, especially from Africa. So, when we come to the UK, to a more advanced country, and then we hear, oh, we have to do screening for breasts and all those things because of our cultural belief and our background, we still have that fear. (Gbemisola – Focus group)

The participant’s account reveals how cultural beliefs surrounding modesty, illness, and mortality profoundly shape her embodied experience of breast cancer screening. She describes the breast as an intensely private part of the body, making the idea of exposing it—even in a clinical context—deeply uncomfortable and culturally transgressive, particularly for women from Nigerian and wider African backgrounds. Hermeneutically, the participant interprets cancer through a culturally and socially construed lens in which the diagnosis is equated with a *“death sentence,”* a belief that transforms screening from a preventive act into a frightening confrontation with mortality. This meaning-making extends to the idea that even knowing one’s diagnosis could *“kill the person,”* reflecting a limited understanding of the disease where awareness itself becomes dangerous and emotionally overwhelming. Ideographically, the participant situates these fears within her own migration journey, noting that even after moving to the UK, these cultural narratives persist, shaping her personal hesitation and the collective anxieties noted among the Black community. Her account illustrates how deeply rooted cultural scripts about the body, illness, and fate continue to influence health behaviours, revealing screening avoidance not only as a product of ignorance about the disease and its management but as a culturally meaningful attempt to protect oneself from fear, shame, and existential threat.

### Tailored communication as a catalyst for screening uptake

In this theme, participants view culturally sensitive communication as essential for overcoming fear, stigma, and uncertainty surrounding breast cancer screening. They emphasise the need for clear, structured information, relatable storytelling, and campaigns that reflect African cultural values and address community-specific concerns. By placing messages in familiar social spaces and using trusted media platforms, participants believe that culturally grounded interventions can normalise screening, reduce emotional barriers, and encourage earlier engagement with breast health services. Talking about this, one participant said:

So, I think that maybe they probably have a system, if they have a system like that for breast cancer training and you know just send us a letter with all the information that we need, that might just help us to be able to go for it. (Alero – Focus group).

The participant’s account highlights a desire for clearer, more structured communication as a way of reducing the uncertainty and emotional burden associated with breast cancer screening. Phenomenologically, she expresses a sense of hesitation rooted in not knowing what to expect, suggesting that the absence of accessible information contributes to anxiety and avoidance. Hermeneutically, her emphasis on *“a system”* and receiving “a letter with all the information” reflects an interpretative process in which knowledge becomes a form of reassurance—something that transforms screening from a vague, intimidating idea into a manageable and legitimate health action. As such, the participant situates this need within her own experience, implying that culturally or personally, formal communication from trusted institutions carries authority and helps counteract fear or misinformation. The participant account illustrates how practical, structured guidance is not merely logistical but emotionally meaningful, offering a sense of clarity and control that can support screening uptake. Another participant also notes:

I think it’s important to address fear and stigma directly. And also, the campaign should help normalise conversations about breast cancer and also show that early detection can lead to better outcome instead of just making it seem like a death sentence. And another thing that I think should be put into consideration is the use of storytelling and real experiences. Yes, because using real stories or relatable scenarios in the artwork can make the message more powerful and emotional. And it also helps people take it seriously. Where the campaign is shown, where the campaign is also shown truly matters. It should be placed in places where people already engage with like social media, community centres, salons, churches, and I think also public transport. (Fiberisma – Focus group)

The participant’s account reflects a desire for breast cancer campaigns that directly confront fear and stigma, revealing how these emotional and social barriers shape their lived experience of health messaging. In making sense of the participant experience, she emphasises the need for campaigns that *“normalise conversations,”* suggesting that silence and taboo create an atmosphere of anxiety in which breast cancer feels like a *“death sentence.”* The participant calls for storytelling and *“real experiences”*, highlighting how emotionally resonant narratives help individuals connect with the issue on a human level, making the message feel authentic rather than abstract. Hermeneutically, the participant interprets effective communication as reshaping cultural meanings—transforming cancer from a symbol of fatalism into one of hope and early detection. In addition, the participant appears to situate her reflections within her own social world, noting that campaigns must appear in familiar, everyday spaces such as salons, churches, community centres, and public transport, where their community naturally gathers. The participant account illustrates how health communication is not simply about information delivery but about engaging cultural values, emotional realities, and community spaces in ways that make screening feel relevant, safe, and socially acceptable. Another participant also said:

Media campaign intervention is something that I will feel comfortable to encourage me to engage in early breast cancer screening uptake. The campaign intervention can target radio or social media platforms but is encouraged to reflect the cultural values of us as Africans, including our concerns and fears and the procedures to go for breast cancer screening to encourage our uptake. I will also suggest like a one-hour online workshop that educates in breast cancer and how to check as well. (Tanashae – Individual interview)

The participant’s account highlights how culturally attuned media campaigns can play a crucial role in shaping their emotional readiness to engage in breast cancer screening. Phenomenologically, they express a desire for interventions that feel safe, familiar, and non−threatening, suggesting that culturally resonant messaging reduces the anxiety and uncertainty that often accompany screening decisions. Her emphasis on campaigns that *“reflect the cultural values of us as Africans”* reveals an interpretative process in which health information appears only meaningful when it acknowledges community−specific fears, beliefs, and lived realities. The participant, therefore, appears to view culturally sensitive communication as a way of transforming screening from something foreign or intimidating into something relatable and legitimate. Idiographically, the participant situates this need within her own experience, noting that platforms such as radio and social media—spaces already embedded in their daily life—serve as trusted channels through which health messages can be received and internalised. The account also illustrates how culturally grounded media interventions are not merely informational tools but emotional and social bridges that help individuals feel understood, respected, and more willing to participate in early screening.

### Trauma, mistrust, and call for psychosocially safe screening pathways

A notable theme captured in the participants’ lived experiences is how past traumatic healthcare experiences, emotional vulnerability, and structural disadvantage such as perceived racial inequities in the NHS shape Black women’s fears around breast cancer screening. Participants emphasise that without trauma-informed communication and robust psychosocial support, screening feels unsafe and potentially re-traumatising. Their narratives reveal deep mistrust rooted in previous perceived neglect within the NHS, alongside a sense of being socially and economically marginalised. For these women, culturally sensitive, trauma-aware care is not optional but essential for making screening feel emotionally secure, respectful, and accessible. Talking about this, one participant said:

One thing I would like to add is the need for psychosocial intervention that could address the depression, anxiety and trauma that occurs both before screening and eventually if a diagnosis is detected. If one is aware that all this support is available to one as a Black woman, then screening become a seamless process. Otherwise, it is practically difficult to go for screening as a Black woman where you know you are already at a disadvantageous position financially, economically and even when it comes to access to care from the NHS in England and Wales. I had a pregnancy that eventually led to a miscarriage, and I was treated poorly in one of the NHS hospitals in Middlesbrough. They just left me like that with very little support and after some time I later went to have a scan in a private centre and by then it was too late. What then happens if one then goes for breast cancer screening? So that is my fear. (Tanashae – Individual Interview)

The participant’s account reveals how psychosocial vulnerability, past trauma, and existing systemic disadvantage shape her lived experience of breast cancer screening. In making sense of the participant’s lived experience, she appears to describe screening as emotionally fraught, noting that depression, anxiety, and trauma can arise *“before screening and eventually if a diagnosis is detected,”* suggesting that the process itself feels psychologically overwhelming. The participant narrative also appears to convey a longing for support systems that could make screening *“a seamless process,”* highlighting how the absence of such care intensifies fear and avoidance. Hermeneutically, the participant interprets her reluctance through the lens of previous negative encounters with the healthcare system, particularly the traumatic miscarriage experience in which she seems to feel *“treated poorly”* and abandoned by NHS staff. This past event becomes a symbolic template for how the participant imagines future care will unfold, shaping her meaning-making around screening as potentially unsafe or neglectful. Idiographically, the participant situates her fears within her identity as a Black woman who feels *“already at a disadvantageous position financially, economically and even when it comes to access to care,”* revealing how personal history, racialised experiences, and systemic inequities intersect to produce deep mistrust. The participant’s account appears to illustrate how screening avoidance is not simply fear of illness but a protective response to anticipated emotional harm, inadequate care, and the possibility of reliving past trauma within a system perceived as unresponsive to their needs. Another participant also said:

Another thing I want to add is being aware of like trauma informed care. Because trauma is like a really big thing, you know, people’s past experience, negative experience. So, you need to have like an awareness of trauma informed principle on how to like care for someone that eventually diagnosed with breast cancer, how to pass the information to them and how to even care for them. It’s very, very important in terms of intervention as well. It’s very important when you are dealing with patients that have been diagnosed with disease. (Gbemisola – Focus group)

The participant’s account foregrounds the centrality of trauma-informed care in shaping how individuals experience breast cancer screening and subsequent treatment. In making sense of her lived experience, the participant describes trauma as *“a really big thing,”* signalling that past negative experiences profoundly influence how healthcare encounters are felt in the body and interpreted emotionally. Her emphasis on *“how to pass the information”* and *“how to even care for them”* reflects a deep sensitivity to the relational and communicative aspects of care, suggesting that the manner in which news is delivered can either retraumatise or support the patient. Hermeneutically, the participant interprets trauma-informed principles as essential to restoring trust, implying that without such approaches, screening and diagnosis may be experienced as threatening or unsafe. The participant appears to view trauma not as an isolated event but as a lens through which future healthcare interactions are understood, shaping expectations of compassion, respect, and emotional safety. As such, the participant situates these reflections within her own awareness of community experiences, highlighting how trauma—whether personal or collective—intersects with cultural identity and past encounters with healthcare systems. Their account illustrates how trauma-informed care is not merely an added layer of support but a fundamental requirement for ensuring that Black women feel protected, understood, and able to engage with screening and treatment without fear of psychological harm.

## Discussion

The purpose of this study was to examine the lived experiences of Black women who are partially or not up to date with breast cancer screening experiences, on the barriers and facilitators to poor early screening uptake in the UK. Based on an interpretative phenomenological analysis of the dataset, several themes emerged, which are discussed in relation to the literature and its implications for policy.

Firstly, the findings revealed a perceived sense of faith-driven reassurance, which appears to intersect with chosen unawareness. Here, faith-driven reassurance is construed as a shield against engaging in breast cancer screening, as faith can be seen as a mechanism of guarantee, safety, and even prevention, whilst normalising the feeling of not engaging in screening. The findings are significant in that the perception that the unknown cannot hurt a person was seen to influence, and in turn, shape how participants negotiate and sustain their narratives, which in turn preclude them from engaging in early breast cancer screening. The finding appears not to be congruent with previous literature (28), which cited a faith-based approach as one of the determinants of poor early screening, whilst undermining the role of selective unawareness, informed by the lack of visible symptoms, as a shield against screening.

A second notable finding from the dataset is stigma, which operates as a significant social barrier that shapes how individuals avoid engagement with breast cancer early screening uptake, particularly in close−knit communities where information circulates rapidly, and anonymity is limited. This reflects the idea of communities within the African and Caribbean context, where issues such as cancer are often attributed to spiritual causes and stigmatised. For example, a study by Oyeleke et al. (29) with Nigerian breast cancer patients found that within Nigerian communities, stigmatisation is perpetuated through language, body shaming, negative stereotypes, cultural and social factors such as beliefs, power dynamics and norms that reinforce stigmatisation. Similar findings were reported in the Caribbean context (30). Our findings show also that there was a perceived sense that being identified as someone who *“went to screen for breast cancer”* or as a person with *“something wrong”* can lead to avoidance, judgment, and subtle forms of social exclusion. The accounts in our study reflect Goffman’s (31) notion of spoiled identity, in which a person becomes discredited once a potentially stigmatising attribute becomes known. This is reinforced by Link and Phelan’s (32) framework, which explains how labelling, stereotyping, and separation culminate in discrimination and status loss. The fear expressed by participants in our study—of being *“looked at weird”* or treated *“somehow”—*aligns with Corrigan and Watson’s (33) concept of anticipated stigma, which often deters individuals from accessing preventive services such as breast cancer screening. Empirical studies similarly show that women may avoid screening not due to the procedure itself but because of the social consequences of being associated with illness (34). In tightly connected social networks, these fears intensify because reputational narratives spread quickly, and social norms are strongly enforced (35). In essence, these insights highlight how stigma is not merely an interpersonal issue but a socially produced barrier that undermines early detection efforts and reinforces health inequalities.

A third key finding is how cultural beliefs surrounding modesty and fatalistic perceptions of illness play a significant role in shaping women’s engagement with breast cancer screening, particularly among African and Nigerian communities. Our findings showed that the breast was construed as a *“very private part of the body,”* noting that cultural norms discourage exposing it, even to the person who’s going to examine it. This reflects findings in the literature showing that modesty norms, gendered expectations, and discomfort with male healthcare providers can reduce screening uptake among African and Black women (34). Alongside modesty, deeply rooted cancer fatalism—captured in the belief that a diagnosis is a death sentence further discourages participation in screening. Such fatalistic beliefs are well−documented barriers to early detection, with studies showing that individuals who view cancer as inevitable or untreatable are significantly less likely to engage in preventive behaviours (36). Importantly, participants noted that these cultural beliefs persist even after migration to the UK, demonstrating that health behaviours are shaped not only by the current context but also by enduring cultural frameworks carried across borders. Thus, the insights highlight how modesty norms and cancer fatalism operate as significant culturally embedded barriers that undermine screening participation and contribute to delayed diagnosis among African women in diaspora communities.

Fourthly, our study found that addressing fear, stigma, and cultural beliefs through structured communication and culturally resonant messaging appears essential for improving breast cancer screening uptake. The participant’s call for a clear system of messaging with all the information needed reflects evidence that organised reminder systems and accessible educational materials appear to significantly increase screening participation, particularly among underserved groups (37). At the same time, the emphasis on campaigns that normalise conversations, counter fatalistic beliefs, and use storytelling aligns with research showing that narrative−based interventions enhance emotional engagement, reduce stigma, and improve health behaviour change (38). Placing campaigns in trusted community spaces—such as churches, salons, and social media—also echoes findings that health messages are more effective when delivered through familiar, culturally meaningful channels (39). These insights appear to highlight that improving screening uptake requires both system−level communication strategies and culturally grounded public health messaging that directly address fear, stigma, and misinformation.

Finally, another finding was the extent to which past traumatic healthcare experiences shaped participants’ fears around screening and bred mistrust. Accounts of neglect, poor communication, and emotional abandonment—particularly during pregnancy loss—appear to create a pervasive mistrust of the NHS. This aligns with literature documenting racial disparities in maternity care and the disproportionate trauma experienced by Black women in UK healthcare settings (40). Participants interpreted these experiences as indicative of how they might be treated if diagnosed with breast cancer, reinforcing avoidance behaviours. The call for trauma−informed care reflects growing recognition that trauma shapes how individuals engage with healthcare and that trauma−informed principles can improve trust, communication, and emotional safety (17). For these women, psychosocial support was not an optional add−on but a prerequisite for feeling safe enough to participate in screening. As such underscoring the need for care that is devoid of racial prejudice or bias to encourage trust and screening uptake.

### Limitations and strengths

The study acknowledges some limitations, including that its findings are based on a small, self−selecting sample, which limits the extent to which the experiences can be generalised beyond the participants. Another limitation is its focus on Black women, and as such, the findings might not be generalisable to other ethnic minority groups, such as South Asian women in the United Kingdom, who might have different experiences compared to our study population. A further limitation, and in line with reflexivity, is the inclusion of two participants with lived experience, whose views may introduce bias or influence. However, this was mitigated through an iterative process with the research team to avoid the risk of bias.

Despite the limitations, this study offers an original and significant contribution by applying Interpretative Phenomenological Analysis to illuminate the deeply personal, culturally embedded, and trauma−shaped experiences that underpin Black women’s low engagement with breast cancer screening. By prioritising lived experience over assumptions embedded in mainstream public health discourse, the study provides a rigorous, idiographic account of how cultural modesty, fatalism, mistrust, and systemic inequities converge to shape screening avoidance. The methodological rigour of IPA—through close, interpretative engagement with participants’ meaning−making—allows the study to reveal nuanced psychological and sociocultural dynamics that are often overlooked in quantitative research, offering insights that can meaningfully inform more culturally responsive and trauma−informed screening policies and interventions.

### Implications for practice

The findings from the present study hold significant implications for policy in that they highlight an urgent need for breast cancer screening policies that explicitly address cultural, psychosocial, and systemic barriers faced by Black women. Healthcare professionals including third sector organisations are encouraged to prioritise the development of tailored communication strategies, ensuring that screening information is accessible, community−centred, and reflective of diverse cultural values. Integrating trauma−informed principles into national screening guidelines is essential, given the profound impact of past negative healthcare experiences on screening avoidance. Policies are also encouraged to mandate equitable access to psychosocial support—before, during, and after screening—to rebuild trust and reduce the emotional burden associated with early detection. Finally, addressing systemic inequities within the NHS, including disparities in communication, continuity of care, and patient safety, is critical for creating screening pathways that Black women can engage with confidently and without fear.

Collectively, our study’s findings suggest that improving breast cancer screening uptake among Black women requires interventions that are culturally grounded, emotionally attuned, and trauma-informed. Clear, structured communication, community-based messaging, and psychosocial support must be integrated into screening pathways. Healthcare professionals are also encouraged to be trained in cultural humility and trauma-informed practice to rebuild trust and ensure that Black women feel respected, understood, and emotionally safe.

## Conclusion

In conclusion, the present study provides a nuanced and in-depth understanding of the factors shaping Black women’s poor engagement with breast cancer screening, revealing how cultural beliefs, emotional vulnerability, trauma, and structural inequities such as unequal access to healthcare intersect to influence screening behaviour. Through the interpretative depth of IPA, the research illuminates the lived realities behind statistical disparities, showing that screening avoidance appears rooted in culturally embedded meanings, mistrust shaped by past harm, and the absence of trauma−informed, culturally responsive care. By centring Black women’s voices, this study challenges existing screening pathways and highlights the urgent need for interventions that embed cultural identity, rebuild trust, and provide psychosocial safety. Ultimately, the findings underscore that improving screening uptake requires awareness campaigns alongside systemic change that recognises and responds to the complex lived experiences of Black women.

## Data Availability

The original contributions presented in the study are included in the article. Further inquiries can be directed to the corresponding author.

## References

[1] World Health Organization . Breast cancer (2025). Available online at: https://www.who.int/news-room/fact-sheets/detail/breast-cancer (Accessed June 23, 2026).

[2] National Institute for Health and Care Excellence (NICE) . Breast cancer – managing FH: How common is it? (2024). Available online at: https://cks.nice.org.uk/topics/breast-cancer-managing-fh/background-information/prevalence/ (Accessed June 23, 2026).

[3] Breast Cancer UK Facts and Figures . (2025). Available online at: https://www.breastcanceruk.org.uk/about-breast-cancer/facts-and-figures/#:~:text=There%20are%20around%2056%2C000%20new%20cases%20of,of%20breast%20cancers%20were%20in%20women%20over (Accessed June 23, 2026).

[4] Breast Cancer Now . Overcoming barriers to breast cancer screening faced by the black African community in the UK (2026). Available online at: https://breastcancernow.org/our-research/research-centres-and-projects/individual-research-projects/overcoming-barriers-to-breast-cancer-screening-faced-by-the-black-african-community-in-the-uk (Accessed June 23, 2026).

[5] Breast Cancer UK Ethnicity and breast cancer risk . (2025). Available online at: https://www.breastcanceruk.org.uk/about-breast-cancer/ethnicity-and-breast-cancer-risk/ (Accessed June 23, 2026).

[6] Aliu AE Kerrison RS Marcu A . A systematic review of barriers to breast cancer screening, and of interventions designed to increase participation, among women of Black African and Black Caribbean descent in the UK. Psycho‐Oncology. (2025) 34:e70093. doi: 10.1002/pon.70093 39891612 PMC11786783

[7] Pfeffer N . Screening for breast cancer: candidacy and compliance. Soc Sci Med. (2004) 58:151–60. doi: 10.1016/s0277-9536(03)00156-4 14572928

[8] Eatough V Smith JA . Interpretative phenomenological analysis. In: The Sage Handbook of Qualitative Research in Psychology, vol. 2. London: SAGE. (2017). p. 193–209.

[9] Thomas VN Saleem T Abraham R . Barriers to effective uptake of cancer screening among black and minority ethnic groups. Int J Palliative Nurs. (2005) 11:562–7. doi: 10.12968/ijpn.2005.11.11.20096 16471043

[10] Jones RH Casbard A Carucci M Cox C Butler R . Fulvestrant plus capivasertib versus placebo after relapse or progression on an aromatase inhibitor in metastatic, oestrogen receptor-positive breast cancer (FAKTION): a multicentre, randomised, controlled, phase 2 trial. Lancet Oncol. (2020) 21(3):345–57. 10.1016/S1470-2045(19)30817-4 PMC7052734 32035020

[11] Webber C Jiang L Grunfeld E Groome PA . Identifying predictors of delayed diagnoses in symptomatic breast cancer: a scoping review. Eur J Cancer Care. (2017) 26(2):e12483. 10.1111/ecc.12483 26950652

[12] Ajit M . Understanding the impact of culturally adapted health education on breast and cervical cancer screening among American Indian and Alaska native women: A Scoping Review. (Masters thesis). Baltimore, Maryland: Johns Hopkins University. (2025).

[13] Schoueri-Mychasiw N Campbell S Mai V . Increasing screening mammography among immigrant and minority women in Canada: a review of past interventions. J Immigrant Minority Health. (2013) 15:149–58. doi: 10.1007/s10903-012-9612-8 22466249

[14] Wu TY Lin C . Developing and evaluating an individually tailored intervention to increase mammography adherence among Chinese American women. Cancer Nurs. (2015) 38:40–9. doi: 10.1097/ncc.0000000000000126 24621965 PMC4161668

[15] Murphy ST Frank LB Chatterjee JS Moran MB Zhao N Amezola de Herrera P . Comparing the relative efficacy of narrative vs nonnarrative health messages in reducing health disparities using a randomized trial. Am J Public Health. (2015) 105(10):2117–23. 10.2105/AJPH.2014.302332 PMC4566521 25905845

[16] Lacobucci G . Most Black people in UK face discrimination from healthcare staff, survey finds. (2022) 378:o2337. 10.1136/bmj.o2337 36167411

[17] Sweeney A Filson B Kennedy A Collinson L Gillard S . A paradigm shift: relationships in trauma-informed mental health services. BJPsych Adv. (2018) 24:319–33. doi: 10.1192/bja.2018.29 30174829 PMC6088388

[18] Bolarinwa OA Holt N . Barriers to breast and cervical cancer screening uptake among Black, Asian, and Minority Ethnic women in the United Kingdom: evidence from a mixed-methods systematic review. BMC Health Serv Res. (2023) 23:390. doi: 10.1186/s12913-023-09410-x 37087506 PMC10122823

[19] Bryman A . Social Research Methods. Oxford: Oxford University Press (2016).

[20] Nightingale DJ Cromby J . Social constructionism as ontology: Exposition and example. Theory Psychol. (2002) 12:701–13. doi: 10.1177/0959354302012005901

[21] Kitzinger J . Qualitative research: introducing focus groups. BMJ. (1995) 311:299–302. doi: 10.1136/bmj.311.7000.299 7633241 PMC2550365

[22] Githaiga JN . Methodological considerations in utilization of focus groups in an IPA study of bereaved parental cancer caregivers in Nairobi. Qual Res Psychol. (2014) 11:400–19. doi: 10.1080/14780887.2014.933918 37339054

[23] Smith JA Flowers P Larkin M . Sage. United States: Thousand Oaks (2009). doi: 10.2307/40136306

[24] Palmer JR Zirpoli G Bertrand KA Battaglia T Bernstein L Ambrosone CB . A validated risk prediction model for breast cancer in US Black women. J Clin Oncol. (2021) 39:3866–77. doi: 10.1200/jco.21.01236 34623926 PMC8608262

[25] Ike TJ Jidong DE Ike ML Francis C Ayobi EE . Reintegration of former Boko Haram members and combatants in Nigeria: An interpretative phenomenological analysis of community members’ experiences of trauma. Third World Q. (2022) 43:2811–29. doi: 10.1080/01436597.2022.2109459 37339054

[26] Ike TJ . ‘Its’ like being a Christian is tantamount to victimisation’: A Qualitative study of Christian experiences and perceptions of insecurity and terrorism in Nigeria. Cogent Soc Sci. (2022) 8:2071032. doi: 10.1080/23311886.2022.2071032 37339054

[27] Jidong DE Husain N Francis C Murshed M Roche A Ike TJ . Mental health experiences of mothers in Jos, Nigeria: An interpretative phenomenological analysis. SAGE Open Med. (2021) 9:2050312120970714. doi: 10.1177/2050312120970714 33889409 PMC8040383

[28] Sage SK Hawkins-Taylor C Crockett RA Balls-Berry JE . Girl, just pray…: Factors that influence breast and cervical cancer screening among Black women in Rochester, MN. J Natl Med Assoc. (2020) 112:454–67. doi: 10.1016/j.jnma.2019.02.006 30935680

[29] Oyeleke JO Okhuosi RE Ayandipo OO . A critical discourse analysis of the language of stigmatization of breast cancer patients in Nigeria. Health Communication. (2025) 40:3048–67. doi: 10.1080/10410236.2025.2492216 40257117

[30] Gil LDC Marín MC Márquez Vila IA Rincón EHH . Barriers to accessing post-mastectomy breast reconstruction in Latin America and the Caribbean: A Scoping Systematic Review. J Racial Ethnic Health Disparities. (2026), 1–12. doi: 10.1007/s40615-026-02955-7 41965487

[31] Goffmann E . Stigma: The Management of Spoiled Identity. Harmdonsworth: Penguin (1963).

[32] Link BG Phelan JC . Social conditions as fundamental causes of disease. J Health Soc Behav. (2001) 35:80–94. doi: 10.2307/2626958 7560851

[33] Corrigan PW Watson AC . Understanding the impact of stigma on people with mental illness. World Psychiatry. (2002) 1:16. doi: 10.1016/b978-0-12-397045-9.00170-1 16946807 PMC1489832

[34] Musa J Achenbach CJ O’Dwyer LC Evans CT McHugh M Hou L . Effect of cervical cancer education and provider recommendation for screening on screening rates: A systematic review and meta-analysis. PLoS One. (2017) 12:e0183924. doi: 10.1371/journal.pone.0183924 28873092 PMC5584806

[35] Pescosolido BA Martin JK . The stigma complex. Annu Rev Sociology. (2015) 41:87–116. doi: 10.1146/annurev-soc-071312-145702 26855471 PMC4737963

[36] Ogedegbe G Cassells AN Robinson CM DuHamel K Tobin JN Sox CH . Perceptions of barriers and facilitators of cancer early detection among low-income minority women in community health centers. J Natl Med Assoc. (2005) 97:162. 15712779 PMC2568778

[37] Everett T Bryant A Griffin MF Martin-Hirsch PP Forbes CA Jepson RG . Interventions targeted at women to encourage the uptake of cervical screening. Cochrane Database SystRev. (2011) 5:CD002834. doi: 10.1002/14651858.cd002834.pub2 21563135 PMC4163962

[38] Murphy ST Frank LB Chatterjee JS Baezconde-Garbanati L . Narrative versus nonnarrative: The role of identification, transportation, and emotion in reducing health disparities. J Communication. (2013) 63:116–37. doi: 10.1111/jcom.12007 24347679 PMC3857102

[39] Viswanath K Kreuter MW . Health disparities, communication inequalities, and eHealth. Am J Prev Med. (2007) 32:S131–3. doi: 10.1016/j.amepre.2007.02.012 17466818 PMC2043145

[40] Knight D Saleem HT Stockman JK Willie TC . Experiences of Black women in the United States along the PrEP care continuum: a scoping review. AIDS Behav. (2023) 27:2298–316. doi: 10.1007/s10461-022-03960-7 36622485

